# OP75 Impact Of Updated Guidelines On Transcatheter Aortic Valve Implantation Outcomes In Brazil: A Decade-Long Retrospective Study

**DOI:** 10.1017/S026646232510130X

**Published:** 2025-12-29

**Authors:** Marcus Borin, Mariana Michel Barbosa, Carina Rejane Martins, Daniel Pitchon dos Reis, Geraldo José Coelho Ribeiro, Júlia Teixeira Tupinambás, Karina de Castro Zocrato, Lélia Maria de Almeida Carvalho, Marcela Pinto de Freitas, Maria da Glória Cruvinel Horta, Mariza Cristina Torres Talim, Sergio Adriano Loureiro Bersan, Silvana Marcia Bruschi Kelles

## Abstract

**Introduction:**

Transcatheter aortic valve implantation (TAVI) has revolutionized the treatment of severe aortic stenosis, compared with more invasive surgical aortic valve replacement (SAVR). Outcomes after device advancements and the landmark 2018 update to TAVI clinical guidelines were analyzed in a 10-year cohort study conducted by a Brazilian health maintenance organization.

**Methods:**

The study analyzed TAVI procedures performed from July 2013 to December 2023 in a Brazilian health maintenance organization. Patients were grouped into two cohorts: those treated before January 2019 and those treated after January 2019, aligning with the implementation of the updated guidelines. Data on patient demographics, procedural details, and clinical outcomes were collected from institutional databases. Primary outcomes included rates of mortality and major complications, while secondary outcomes encompassed rates of procedural success and device-related complications. Statistical comparisons were conducted using chi-square and t-tests, with significance set at p<0.05.

**Results:**

The study included 325 patients, with 56 treated before and 269 after the guideline update. There was no difference in median age (84 years) between the two cohorts, and 58 percent were female. After guideline implementation, a shift to self-expanding valves resulted in reduced rates of pacemaker implantation (28.6 to 18.2%; p=0.03) and stroke (8.9 to 5.6%; p=0.2). The one-year mortality rate decreased from 25 to 14.9 percent, reflecting significant improvements in clinical outcomes. Procedural success rates exceeded 98 percent in both cohorts, demonstrating the reliability of TAVI in routine practice.

**Conclusions:**

The implementation of updated procedural protocols in 2018 led to significant improvements in TAVI outcomes for patients with similar risk profiles. These findings underscore the importance of evidence-based practice and continuous technological advancements in optimizing patient care for severe aortic stenosis. Future studies should focus on long-term outcomes and durability of TAVI devices.

